# Flow Characterization of a Spinner Flask for Induced Pluripotent Stem Cell Culture Application

**DOI:** 10.1371/journal.pone.0106493

**Published:** 2014-10-03

**Authors:** Mohd-Zulhilmi Ismadi, Priyanka Gupta, Andreas Fouras, Paul Verma, Sameer Jadhav, Jayesh Bellare, Kerry Hourigan

**Affiliations:** 1 Division of Biological Engineering, Monash University, Melbourne, Victoria, Australia; 2 Department of Mechanical and Aerospace Engineering, Monash University, Melbourne, Victoria, Australia; 3 Department of Chemical Engineering, Monash University, Melbourne, Victoria, Australia; 4 Department of Chemical Engineering, Indian Institute of Technology Bombay, Mumbai, India; 5 South Australian Research and Development Institute, Rosedale, South Australia, Australia; University of Tampere, Finland

## Abstract

We present detailed quantitative measurement analyses for flow in a spinner flask with spinning rates between 20 to 45 RPM, utilizing the optical velocimetry measurement technique of Particle Image Velocimetry (PIV). A partial section of the impeller was immersed in the working fluid to reduce the shear forces induced on the cells cultured on microcarriers. Higher rotational speeds improved the mixing effect in the medium at the expense of a higher shear environment. It was found that the mouse induced pluripotent stem (iPS) cells achieved the optimum number of cells over 7 days in 25 RPM suspension culture. This condition translates to 0.0984 Pa of maximum shear stress caused by the interaction of the fluid flow with the bottom surface. However, inverse cell growth was obtained at 28 RPM culture condition. Such a narrow margin demonstrated that mouse iPS cells cultured on microcarriers are very sensitive to mechanical forces. This study provides insight to biomechanical parameters, specifically the shear stress distribution, for a commercially available spinner flask over a wide range of Reynolds number.

## Introduction

Neurodegenerative disorders such as Alzheimer’s disease, brain and spinal injuries, and Parkinson’s disease affect more than 6 million people in North America. The number of cases can be translated to over 150 billion dollars in healthcare costs each year [1]. Methods for repairing and replacing damaged or absent tissues and organs have been of major interest to relieve the immense burden in healthcare expenditure and also to improve quality of human life.

Since the development of pluripotent stem cells, such as embryonic stem (ES) cells and induced pluripotent stem (iPS) cells, the focus in regenerative medicine has shifted from organ transplantation to cell therapy. Due to the stem cells’ self-renewing nature and ability to differentiate to various types of cells, the stem cell is the perfect basic material for treatment of degenerative diseases. Scientific research can also benefit from the renewal ability by having a large number of the cells available through the proliferation process.

One of the major shortcomings of stem cell therapy is that it requires a significant number of cells. For example, one to two billion cardiomyocytes are required in order to treat damaged heart tissue after myocardial infarction, and about 1.3 billion insulin-producing β cells are needed to realize insulin independence for diabetes patients [2]. The bioreactor has been used as a device to proliferate cells to larger numbers as well to guide the cells in the differentiation process. As a result, optimizing the bioreactor has been of major research interest [3], [4], [5] to achieve a high cell number required for therapeutic purposes. Bioreactor design for cell culture varies from the perfusion bioreactor [6], [7], [8], the rotating wall bioreactor [9], [10], [11] to the most-commonly used spinner flask bioreactor [12], [13], [14], [15]. It is important that the bioreactor allows control over the characteristics of the serum environment such as pH, oxygen dissolution rate, temperature, nutrient transfer, and mechanical stimulation to ensure repeatability and reproducibility of the cells. However, the optimized condition is highly dependent on the type of the cell. Thus, designing a bioreactor for general cell culture use is a major challenge.

The spinner flask bioreactor is widely used in culture procedure due to its availability, ease of setup and also its ability to provide a homogeneous condition throughout the process. Although it is difficult to design a generic bioreactor for a wide-range of cell types, the spinner flask has been proven to derive a wide range of cells by varying the agitation rates [14], [15], [16], [17], [18]. Niebruegge et al. [17] and Zandstra et al. [15] were able to grow cardiomyocytes at 60 RPM. On top of being able to grow multipotent cells, the spinner flask has been demonstrated to be able to grow pluripotent cells. Mouse and human ES cells were successfully grown in a spinner flask at 80 RPM, as shown by Schroeder et al. [14] and Cameron et al. [16], respectively. Furthermore, the cell yield can be further enhanced by the introduction of microcarriers, which increase the surface area for cell growth as shown in previous studies [19], [20], [21], [22], [23], [24].

There is no specific recipe or protocol for cell culture. The procedure to grow cells is different, depending on the type of cells used. Furthermore, there are no specific guidelines in terms of the rotational speed, chemistry and volume of culture medium. In most studies, the location of the impeller is not mentioned. In order to improve the cell culture system as a whole, it is necessary to outline a specific protocol, which would allow optimization of various aspects of cell culture to be done independently.

Flow characterization and hydrodynamic force are aspects that have been overlooked in the past in developing cell culture protocols as well as the bioreactor designs. As these properties are out of the biologists’ or cell culturists’ area of expertise, most cell studies correlate the number of the cells directly to the stirring rate, without understanding the details of the flow behavior in the reactor. Moreover, the lack of understanding in mechanical interaction with the cells limits the progression of bioreactor technology. Several published studies have attempted to understand the fluid dynamics using experimental and computational methods [25], [26], [27], [28]. However, only a few of these studies examined the importance of hydrodynamic force [25], [27]. Furthermore, some studies used a novel type bioreactor as the focal area of analysis while others focused on the flow around a construct or scaffold, rather than profiling the flow in commercially available bioreactors [27], [29], [30], [31]. Recently, a novel holographic technique was developed to characterize the flow condition in the spinner flask [32]. Furthermore, Gupta et al. [33] systematically investigated the effect of rotational speed on microcarrier structures and cell growth. The understanding of fluid mechanics in bioreactors is necessary to provide insights into enhancing the bioreactor efficiency, thus improving the culture process.

This article describes an experimental approach to characterize the fluid dynamics in a 100 mL Bellco spinner flask. The Bellco spinner flask was chosen for this study due to its common impeller design and its frequent use for biological work. Velocimetry techniques have been known to be able to analyze complex flows in diverse applications. In this study, Particle Image Velocimetry (PIV) is chosen to quantitatively describe the meridional and azimuthal flow profiles in the bioreactor.

PIV is a full field, optical-based measurement technique that has been developed for more than twenty years. The technique statistically measures the displacement of the scattered light between consecutive images, captured by a high-speed camera. The scattered light is produced by tracer particles seeded in the fluid being illuminated by a bright light source, typically a Nd:YAG laser. Assuming the particles follow the fluid flow perfectly, the technique is able to quantitatively characterize the flow with high accuracy through the use of cross-correlation, which provides the distribution of the particle displacement, performed on the discretized images. The highest signal in the cross-correlation map represents the most probable particle displacement between image frames. Knowing the time difference between image frames, the velocity of the flow can be calculated.

This study is aimed at characterizing biomechanical properties associated with cell culture procedures in a commercially available spinner flask to define the design parameters for the bioreactor. It is anticipated that the characterization of frequently used bioreactors would benefit many research groups and, thus, improve the pace in cell protocol and bioreactor design developments. This study outlines extensive shear analysis in a spinner flask at varying rotational speeds, alongside cell culture results with similar experimental parameters.

## Methods

### Imaging Experimental Setup

**Figure 1:**
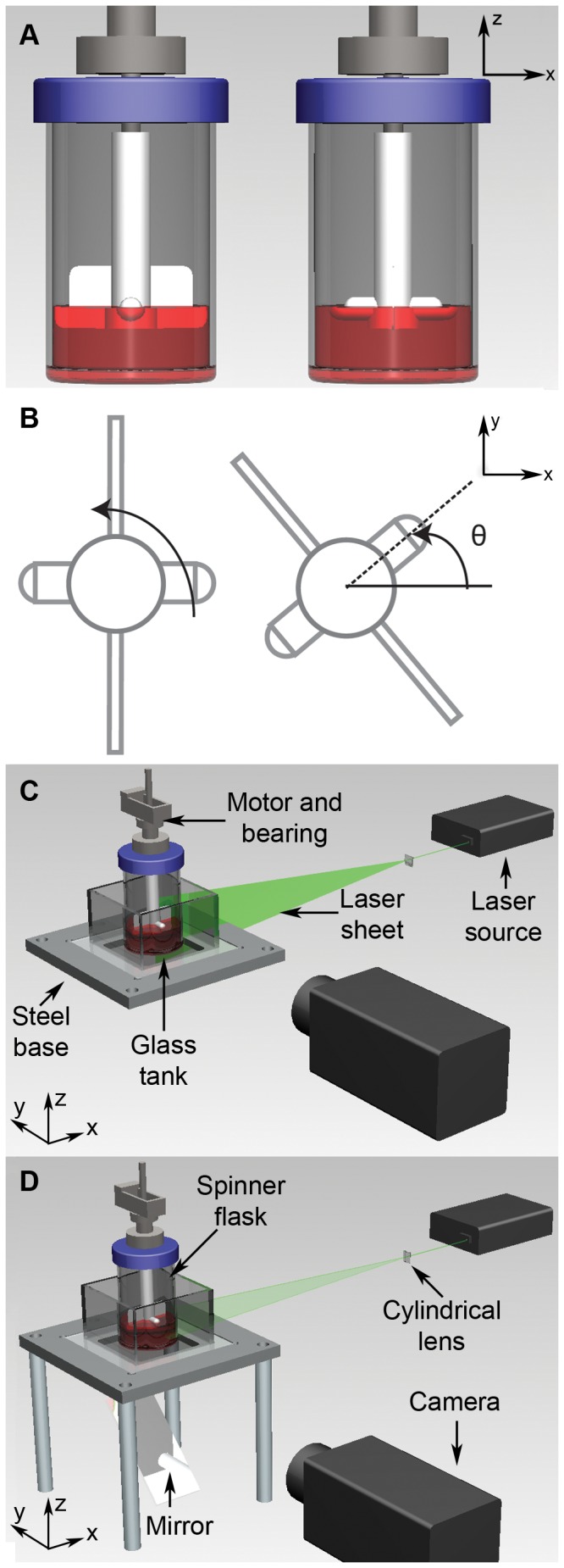
Experimental configuration for PIV measurement in spinner flask bioreactor. A) The impeller consisted of a thin rectangular Teflon sheet and a magnetic stirring bar. The middle height of the magnetic rod was placed at the fluid level throughout the experiment. B) Top view of the impeller. The impeller rotates in counter-clockwise direction throughout the experiment. The spinner flask was placed inside a square tank filled with water to eliminate the lensing effect due to the flask curvature. The working fluid was seeded with 31 µm fluorescent particles and agitated by the impeller driven by a stepper motor. A cylindrical lens was used to create a laser sheet to illuminate the imaging region. The seeded flow was imaged with a high-speed camera (IDT Y4). C) In the meridional plane imaging configuration, the laser sheet was aligned vertically and placed at the center of the flask. D) For measurements in the azimuthal plane, the laser sheet was aligned to the horizontal plane and a mirror was placed underneath the setup to allow view access to the illuminated region.

The study of flow inside a spinner flask, agitated by a rotating impeller, is presented. The experimental layout is shown in Figure 1. A 100 mL microcarrier spinner flask (BellCo Glass Inc, USA), with internal diameter of 55 mm, was filled with 50 mL of distilled water. The water was seeded with fluorescent particles having nominal diameter of 31 µm at 1 mg/mL seeding density and which faithfully follow the flow in the flask. The particles were illuminated by a laser sheet generated by a Nd:YAG laser (Darwin λ = 532 nm) that operates at 40 kHz. For the purpose of this work, the laser was considered to produce a continuous wave illumination. For each measurement, the laser sheet was carefully aligned at the imaging plane in the bioreactor. In order to reduce the lensing effect due to the curvature of the flask wall for the imaging procedure, the flask was placed inside a rectangular tank filled with water. The set-up was mounted on a steel base plate and secured to a precision optical table to eliminate any vibrations.

The flow under study was induced by the rotation of the impeller, driven by a stepper motor (Sanyo Denki America Inc, USA). As shown in Figure 1, the impeller consisted of a flat, rectangular Teflon sheet and a small magnetic stirring bar. In all measurements, the impeller was placed at a height where half of the magnetic rod was immersed in the water as presented in Figure 1A. Such a configuration was chosen to achieve a lower shear environment due to the sensitivity to hydrodynamic forces of the cell types chosen. The motor was run through a motion controller (National Instruments Australia, North Ryde, NSW, Australia), which enabled a maximum of 5.14×10^4^ steps per revolution. To ensure a smooth rotation of the impeller at all speeds, the velocity of the motor was further geared down by a factor of 30. The Reynolds number of the flow was defined as Re = ΩR^2^/ν, with Ω being the angular velocity of the impeller, ν being the kinematic viscosity and R being based on the radius of the flat impeller (R = 25.3 mm).

To keep track of the impeller position throughout the measurements, the angular coordinate system is required (see Figure 1B). The stepper motor was set to turn in a counter-clockwise direction from the top view. A horizontal line to the right of the impeller was chosen as the datum line, as depicted by a solid line in Figure 1B. The angular position of the impeller was defined as the angular distance between the datum line to the centerline of the magnetic rod throughout the manuscript.

The flow was visualized with a high-speed CMOS camera (IDT Y4) fitted with a Nikkor 105 mm f/2.8G lens (Nikon, Japan) with an exposure time of 300 µs. The acquisition rate was set so that each image was captured at one-degree rotation spacing. The camera frame rate in Hertz (Hz) can be calculated by multiplying the rotational speed in RPM by 6. This translates to an acquisition rate ranging from 120 Hz for 20 RPM to 270 Hz for 45 RPM. One set of data consisted of 361 images (360 image pairs). Throughout the experiment, 12 sets of data were taken at each speed for averaging purposes. It was ensured that before any image was captured, the setup was left running until steady-state flow was achieved.

To study the flow in the bioreactor, three main analyses were conducted in the meridional and azimuthal planes – the flow profile in the meridional plane, the flow profile in the azimuthal plane, and the fluid interaction at the bottom surface of the flask. In the meridional plane flow measurement, the laser sheet was placed vertically at the center of the flask (Figure 1C). For azimuthal measurement setting, the laser sheet was aligned horizontally and a mirror was placed underneath the tank. In this setup, the camera was placed to capture images reflected by the mirror, as presented in Figure 1D.

**Figure 2:**
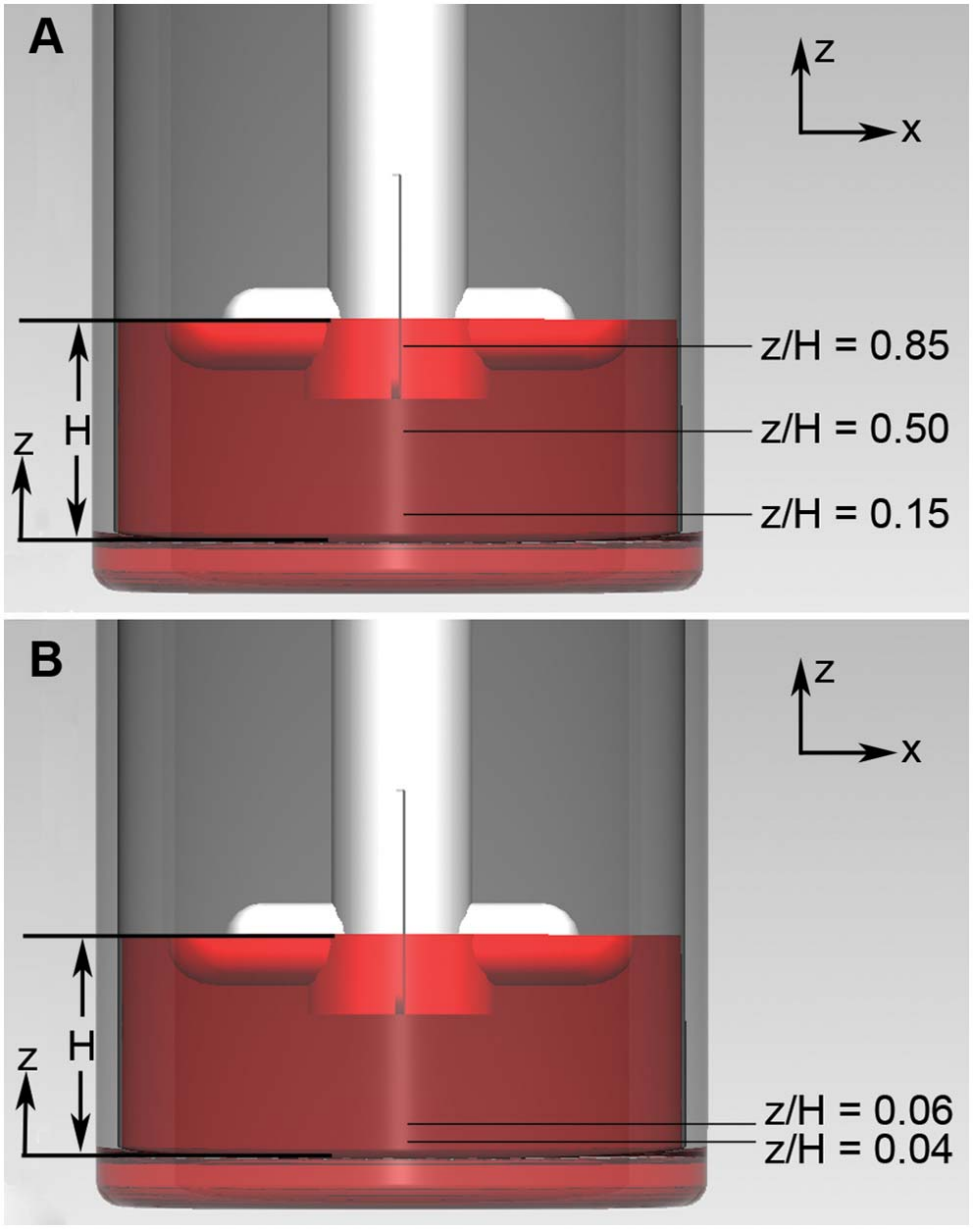
Azimuthal plane measurement locations. A non-dimensional parameter, z/H, is used to show the location of the measurement, where z is the distance from the bottom wall and H is the height of the fluid free surface. In our experiment, H = 19 mm for 50 mL working fluid. A) Three measurements were conducted at z/H = 0.15, 0.50 and 0.85. Velocity, shear stress and vorticity analyses were conducted at each measurement. B) Two measurements were conducted near the bottom wall. A parabolic function was fitted between the measurements at z/H = 0.06 and z/H = 0.04. Then, the velocity gradient near the bottom wall was calculated.

A total of six measurements were recorded at each rotational speed. One measurement was conducted in the meridional plane, three measurements at three different heights were conducted in the azimuthal plane (Figure 2A), and finally two measurements near the bottom wall were done in the azimuthal plane (Figure 2B). By having two measurements near the bottom wall plus the non-slip condition at the wall, an accurate approximation of shear rate was calculated by fitting a parabolic function to the data. The rotational speed was varied between 20 RPM (Re = 1335) and 45 RPM (Re = 3004) at 5 RPM increments.

### Data Processing

Stationary artifacts were removed to improve the data quality. This was done by performing background subtraction using the local temporal average of the image sequence. A maximum vertical deformation of 0.5 mm was seen at the free surface. To eliminate the discrepancy of the datasets, the top of each image was cropped 0.5 mm. Next, a mask file was generated to remove the impeller (in datasets where the impeller was in the imaging plane) and area outside the flask in the computing analysis. The analysis was performed using in-house PIV software, which has been rigorously tested over a number of years [34], [35]. The images were divided into 128×128 pixel interrogation-windows and analysis was conducted at a spacing of 8×8 pixels in x and y directions, respectively. Considering one complete rotation consisted of 360 phases (one degree increment), the results were phase-averaged over the 12-recorded datasets to enhance the accuracy of the measurement. Shear stress, τ, and vorticity, ω, were calculated using the equations shown below,



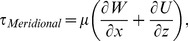
(1)




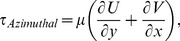
(2)




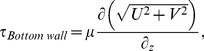
(3)




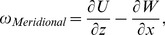
(4)where µ is the dynamic viscosity of water and U, V, W are the velocity components in the x, y, and z directions, respectively. The coordinate system is shown in Figure 1. Due to the no-slip condition at the bottom surface, the in plane velocity at the wall is zero. In this case, only the z derivative is not zero in general at the bottom wall, which results in equation 3.

### Cell Culture Procedure

A parallel cell study was conducted to measure the effect of hydrodynamic force on live mouse OG2 iPS cells, derived according to Tat et al. [36]. Mouse OG2 iPS cells, attached to Cytodex 3 microcarriers, were grown in the spinner flask bioreactor, filled with 50 mL culture medium, in a humidified incubator at 37°C with 5% CO_2_ level for 7 days. Due to the sensitivity of the chosen cells to shear conditions, only a partial section of the impeller was immersed in the culture medium, similar to the imaging configuration. The cells were seeded at a density of 2×10^5^ cells/mL and for the first 24 hours, no agitation was induced in the system to promote cell attachment to the microcarriers. For the next day and onwards, the culture medium was stirred at a specific speed. Throughout the experiments, 50% of the medium was replaced every day. At the end of day 7, viable cells were counted using a haemocytometer after trypan blue staining. The experiments were repeated at various agitation speeds and three repetitions were conducted for each speed. For more details, see Gupta et al. [33], [37].

## Results and Discussion

Flow profiles in the meridional and azimuthal planes are presented. The impeller and zone outside the flask, where no flow existed, were excluded in the PIV calculation. The analysis was conducted on half of the flask, considering the system has a periodic flow. The studies were split into three sections - meridional plane analysis, azimuthal plane analysis and shear profile analysis at the bottom surface of the flask. In the meridional plane, shear stress and vorticity were characterized as overlaid on the velocity profile. On the other hand, for the azimuthal plane analysis, the vorticity calculation was not shown due to the fluid flow behaving like a solid body rotation, producing minimal variance in vorticity.

### Flow Profile in Meridional Plane

**Figure 3:**
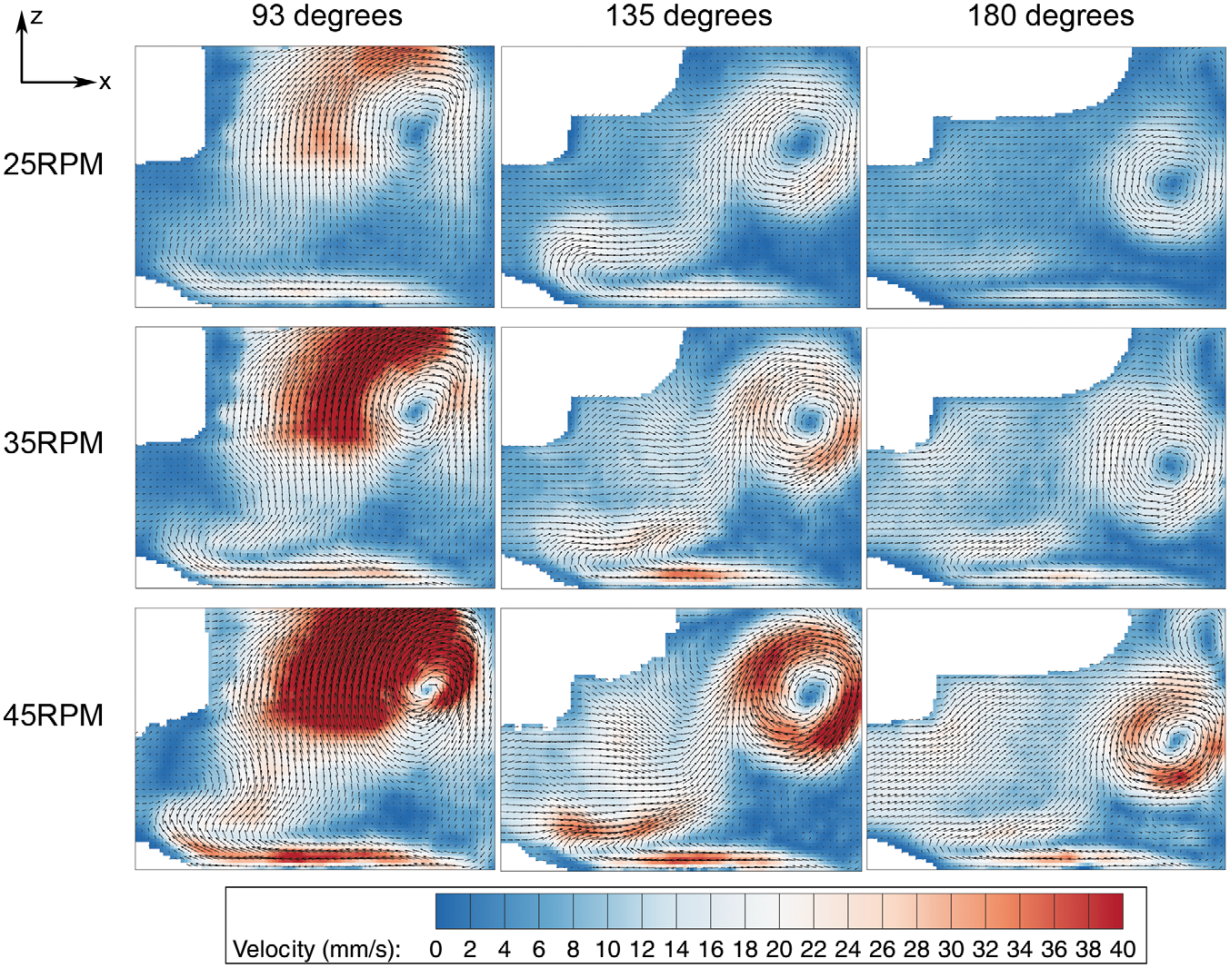
Velocity magnitude contour for flow in spinner flask in meridional plane. The velocity increases with increasing agitation speed. Furthermore, the fluid accelerates radially outwards at highest magnitude behind the flat impeller.

**Figure 4:**
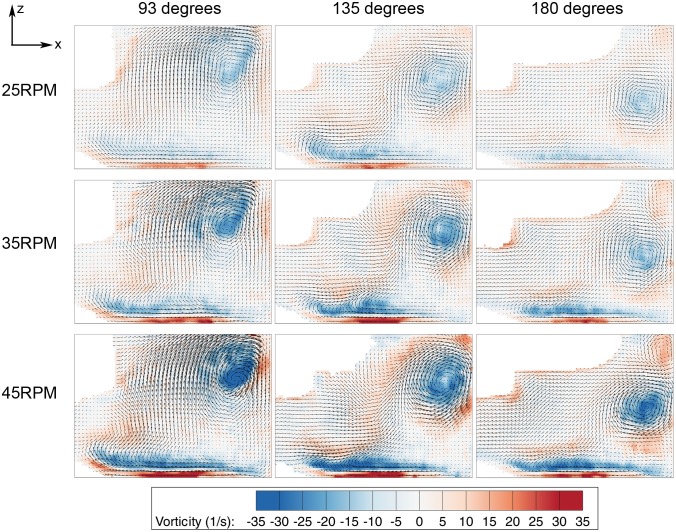
Vorticity evolution at 3 angular positions for 25, 35 and 45 RPM. The main vortex slowly reduces its strength as the plane of measurement shifts from 93 degrees to 135 degrees.

**Figure 5:**
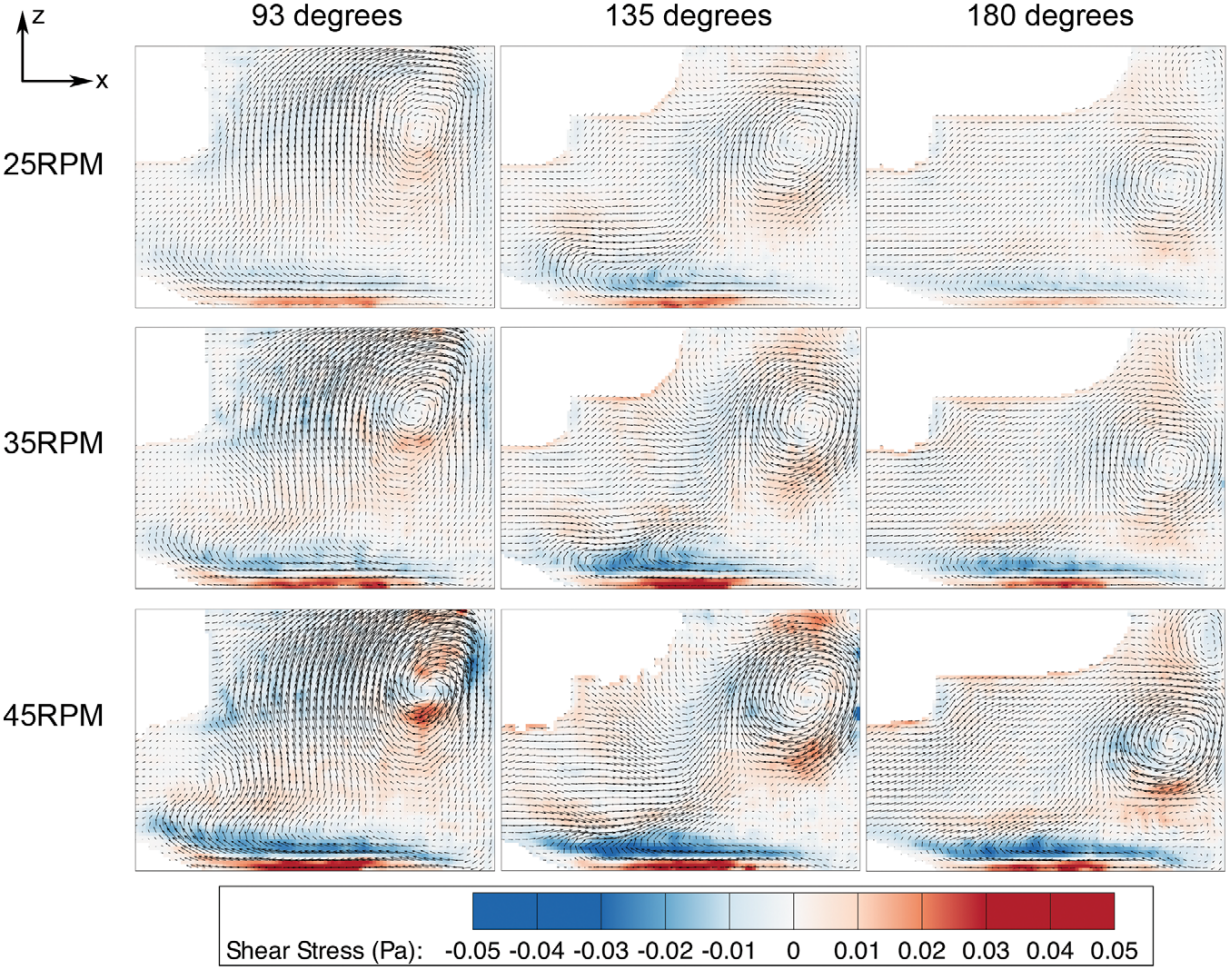
Shear stress distribution in azimuthal plane at varying phases. Significant shear stress can be seen at the bottom wall, caused by a high velocity gradient due to the interaction of the fluid with the wall.

The meridional flow profile at various impeller speeds was characterized. In Figure 3, 4 and 5, the columns and rows show the different rotation phases and rotational speeds, respectively. The contour in Figure 3, 4 and 5 illustrate the magnitude of velocities, vorticity and shear stress, correspondingly, overlaid on the velocity vectors. The impeller rotated into the page in all figures. The white area in the figures are the impeller and small bump that were omitted from the PIV calculation. In these figures, only one quarter (90–180 degrees) is shown to highlight the main feature of the fluid behaviour, as there are very minimal changes in flow characteristics beyond 180 degrees.

Based on Figure 3, it can be seen that the velocity magnitude increases with Reynolds number. As the impeller spins, the fluid is accelerated radially outwards towards the sidewall, flowing downwards to the bottom wall. The fluid then travels inwards and goes up at the center of the flask producing a global clockwise flow. At each spin rate, a similar flow structure is obtained and the flow achieved its maximum near the bottom wall and surrounding impeller area, slightly after the flat impeller passed the imaging plane. However, a slight but distinct structure variance can be noticed at 135 degrees between low and high spin rates. At low spin rate, the fluid circulates smoothly from bottom surface upwards. In contrast, the fluid is being pushed downwards, producing more abrupt change in direction at high spin rate. Furthermore, higher rotational speed produced higher vertical velocity at the center of the rotation compared to lower spin rates. The vertical velocity provides lift for the microcarriers to keep them in suspension throughout the culture procedure. Some particle settlement was noticed around the bump at 20 RPM.

Vorticity is a quantitative, local measure of circulation. Vortices provide a mixing mechanism in the system to ensure a relatively homogeneous concentration of nutrients in the flask. In the analysis we conducted, a positive value of vorticity represents anti-clockwise circulation and vice-versa. Figure 4 depicts the vorticity contours at various speeds and rotation angles. Overall, the vorticity magnitude increases with increasing rotational speed. Additionally, similar to the velocity profile, vorticity peaks in the region downstream of the flat impeller, in this case, at 93 degrees. The main vortex then translates downwards while showing some reduction in magnitude. At the bottom surface, the vorticity grows as the plane of measurement shifts from from 93 degrees to 135 degrees due to the increment of velocity at that particular area. At 180 degrees, there are small vortices rotating in a counter-clockwise direction above and below the main vortex.

**Figure 6:**
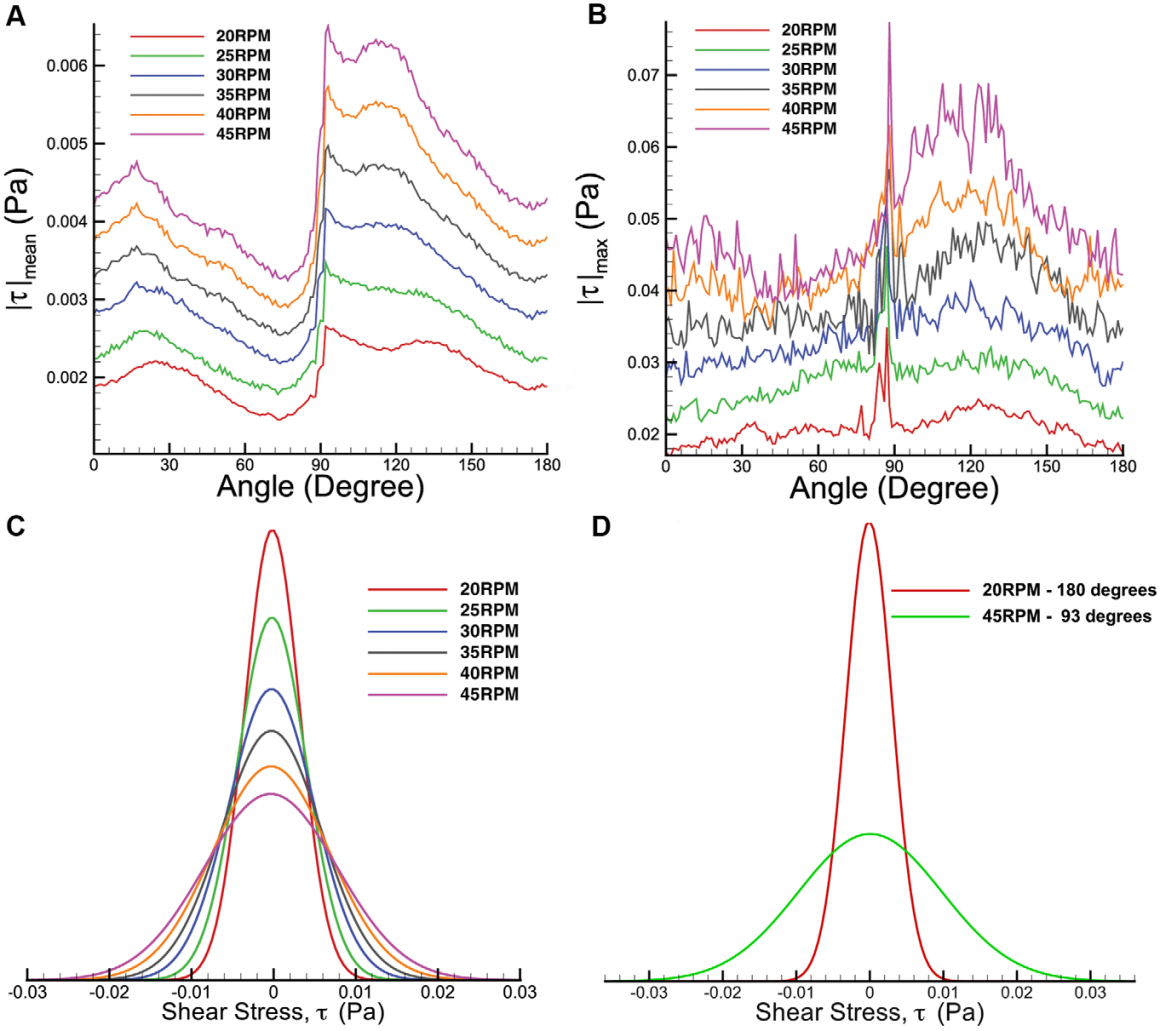
Shear stress distribution for varying rotational phase. A) Mean and B) maximum shear stresses. In both plots, the shear readings spike at 90 degrees, where the flat impeller is in the imaging plane. C) Histogram of shear stress for various speeds between 20 and 45 RPM. D) Shear stress distributions over meridional plane at two extreme cases; 20 RPM at 180 degrees and 45 RPM at 93 degrees.

The shear is relatively high in the region close to the bottom surface, with noticable shear around the vortex as illustrated in Figure 5. To understand the shear profile in the meridional plane, the maximum, mean and distribution of shear stress over the meridional plane were plotted and shown in Figure 6. Only the first 180 degrees are given prominence due to the symmetrical geometry of the impeller. In the mean shear stress plot (Figure 6A), the data shows a similar pattern, but with a linearly increasing magnitude at higher Reynolds numbers. At each spin rate, the highest reading occurs at 90 degrees at the sidewall of the flask. In the maximum shear stress plot in Figure 6B, the measurements are more than 10 times higher than the average shear stress. At each individual spin rate, the shear stress peaks at around 90 degrees, where the highest velocity magnitude is achieved within the rotation phase. The shear stress margin widens at high agitation speeds, which can be seen in the distribution plot in Figure 6C. Additionally, the shear stress distribution for 45 RPM spinning rates specifically at 93 degrees phase angle illustrates wider margin in Figure 6D compared to the overall distribution in Figure 6C at similar speed. The plot demonstrates that the shear stress is significantly higher at 93 degrees. On the other hand, minimal difference is seen at lowest shear stress condition (20 RPM at 180 degrees phase angle) compared to overall shear density plot. The plots at extreme cases shown in Figure 6D highlight the minimum and maximum shear condition over the meridional plane in this experiment.

**Figure 7:**
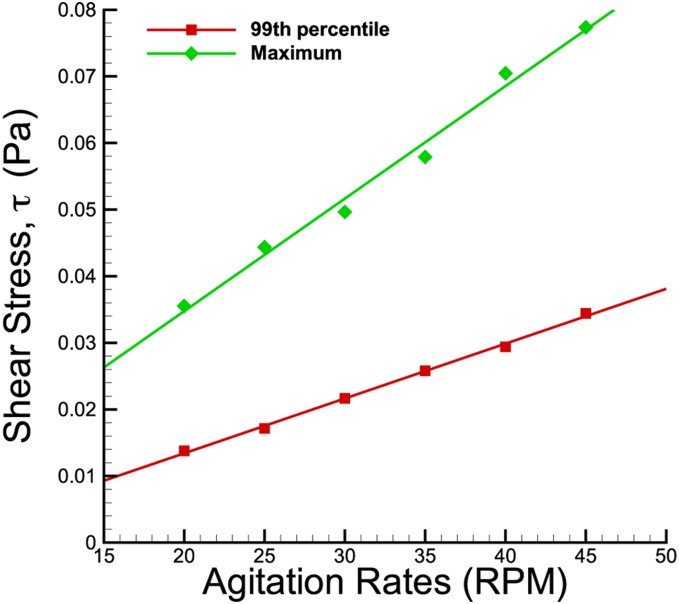
Maximum and 99^th^ percentile shear stress plots in the meridional plane at varying spinning rates. The plots increase linearly with the agitation rates. However, the shear stress magnitudes at 99^th^ percentile are marginally lower than the maximum values at each speed.

The maximum and 99^th^ percentile shear stress magnitude corresponding to the agitation rate are plotted in Figure 7. Both plots show increasing linear relationship to the impeller speed. However, the 99^th^ percentile values are noticably lower than the maximum shear stress. The large discrepancies may have been caused by significant shear gradient around the impeller tip at certain phase angle that contributed to the maximum value.

### Azimuthal Flow Profile

**Figure 8:**
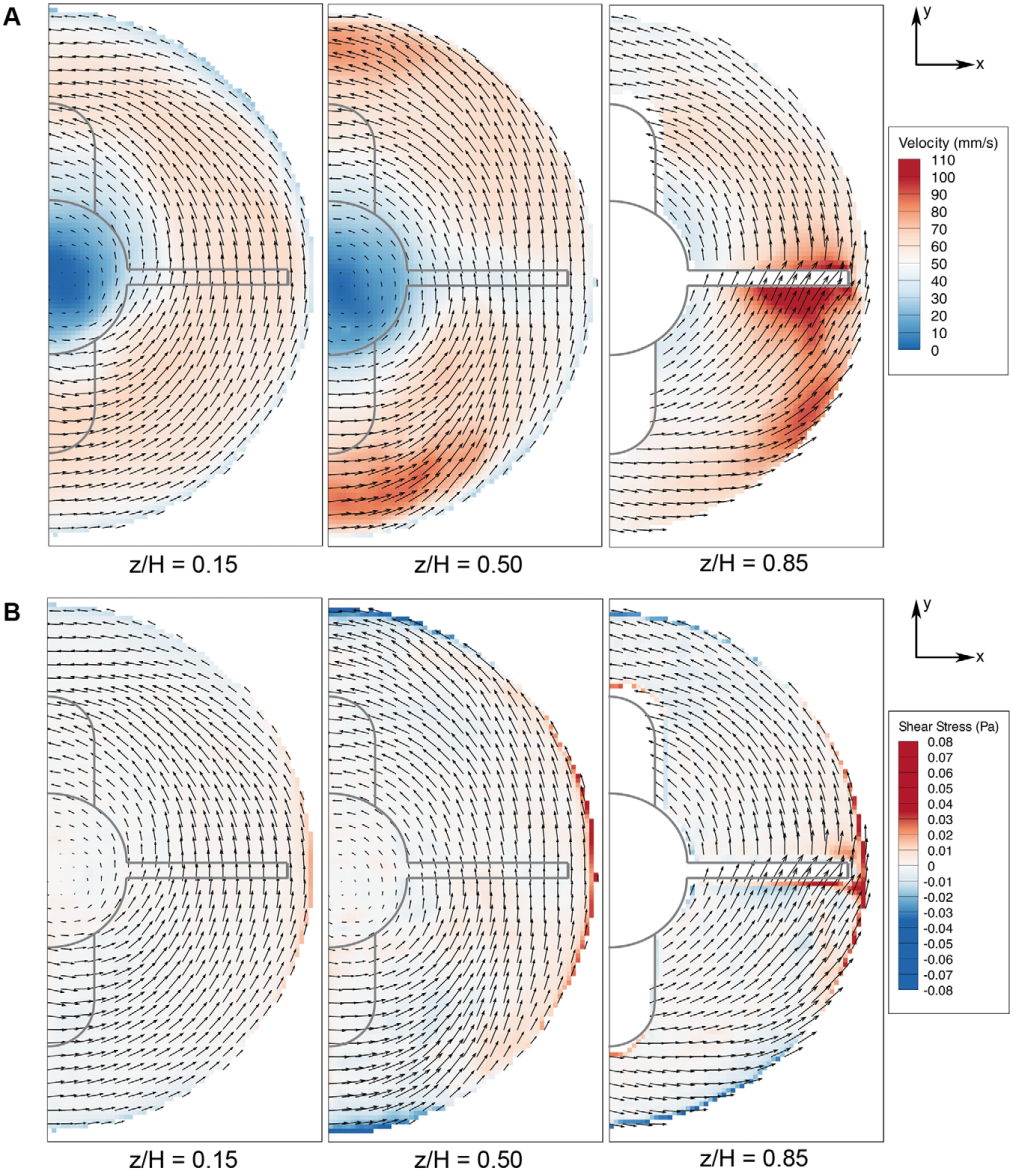
Flow visualization in azimuthal plane. A) Velocity and B) shear stress contours at z/H = 0.15, 0.50 and 0.85. Highest velocity and shear stress magnitudes are achieved around the flat impeller region in the z/H = 0.85 plane. The farthest plane from the impeller shows homogeneous distribution flow characteristics in both velocity and shear plots.

The azimuthal flow is the primary velocity component in the spinner flask. Similarly, velocity and shear are characterized in this plane. The flow is rotating like a solid body rotation, except for a thin boundary layer around the sidewall, where the no-slip condition is enforced, the vorticity is almost constant at all imaging planes. Measurements were conducted at three different heights, z/H = 0.15, 0.50 and 0.85. For the purpose of visualization, only the velocity and shear profiles for 40 RPM (Re = 2670) are shown. As the flow is in steady state, only one phase is shown at each height that highlights the main flow characteristics in Figure 8.

The velocity magnitude is low at the center of rotation at all heights. In general, it can be seen that the flow is mainly in the tangential direction with a minor radial component (Figure 8A). As the imaging plane gets further away from the impeller, the velocity reduces and a more homogeneous velocity distribution is achieved near the bottom surface. At the highest imaging slice, the highest velocity is recorded near the flat impeller. This is due to the fact that the impeller is within the imaging plane at z/H = 0.85. Unlike at the highest imaging plane, in the middle-height section, the high velocity area is near the magnetic rod. As shown previously in the meridional plane characterization, the circulation travels downwards, which contributed to the high value visualised at z/H = 0.50 near the magnetic rod region. The standard deviation of the velocity measurements varies between 8.01 mm/s to 15.62 mm/s within 20 RPM to 45 RPM speed margin. The largest velocity variation (hence standard deviation) within the imaging plane was recorded at the highest imaging plane, where the measurement made is closest to the impeller. The rotating impeller provides the driving force in the flow and therefore creates high-speed region around it. The lowest standard deviation, achieved at z/H = 0.15, showed that the flow is more homogeneous at a plane far from the impeller.

Figure 8B illustrates the shear stress contour at various heights. At each plane, it can be seen that high shear occurs due to the rapid deceleration of the flow because of the no-slip condition at the sidewall. Additionally, the shear stress is highly dependent on the velocity magnitude. The sidewall shear reading is lowest at z/H = 0.15 and increases at the higher planes due to the greater magnitude of velocities. At the 0.85 height ratio, the sharp edges at the flat impeller provide strong force to agitate the fluid but at the same time it produces a significant velocity gradient, which translates to a substantial shear reading.

**Figure 9:**
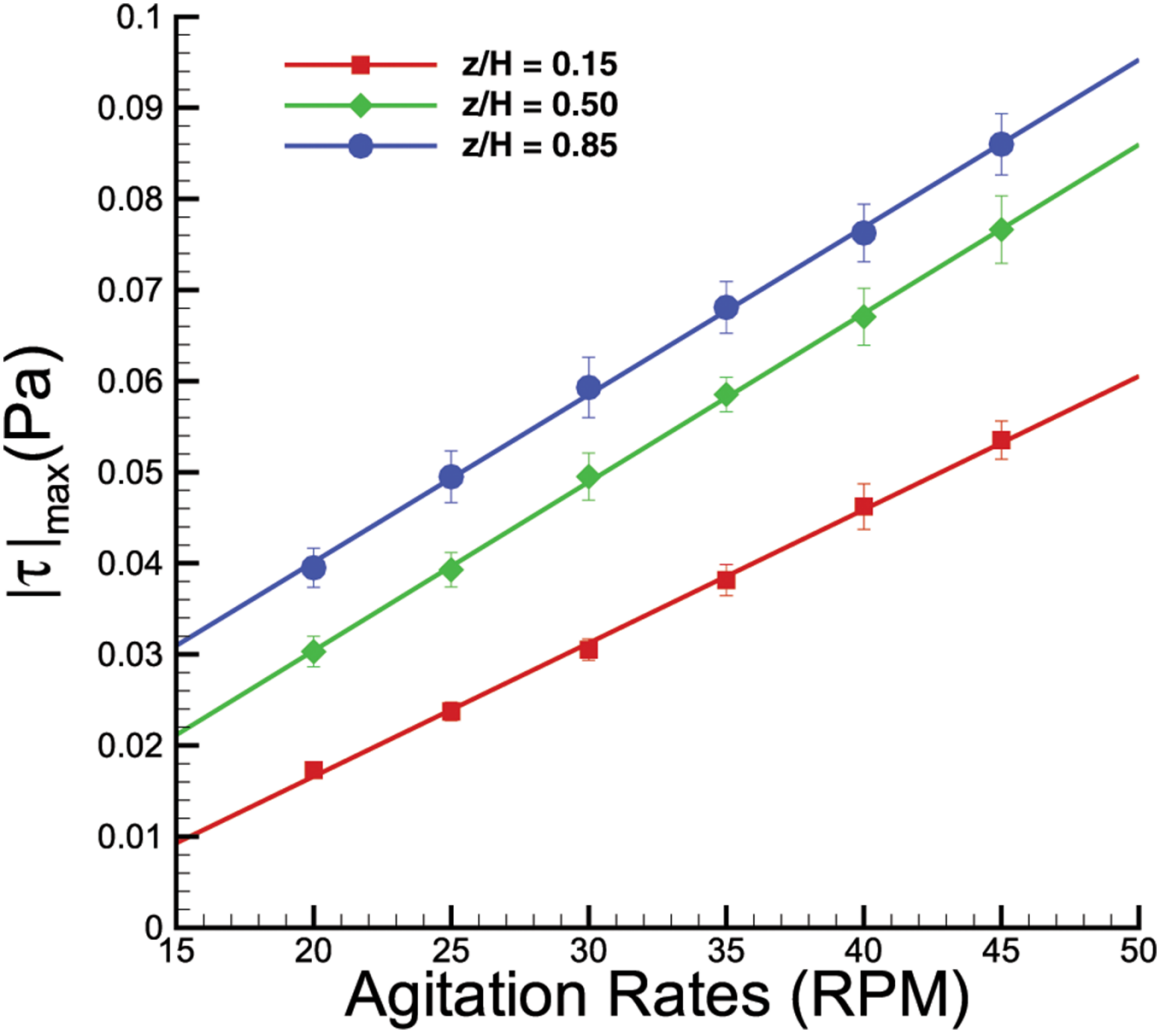
Plots of maximum shear stress when the bioreactor was spun at 20 to 45 RPM. Although at each height the shear varies linearly with the spinning rates, the slopes differ from one to another.

The results are summarized in a graph of maximum shear stress versus agitation rate, as shown in Figure 9. Due to the axisymmetric nature of the flow in the azimuthal plane, shear readings over a full rotation can be further averaged to enhance the accuracy of the analysis. The plots show that the shear stress behaves linearly with agitation speed. Presumably, one could extrapolate the graph to obtain an accurate approximation of the shear magnitude for spinning rates outside the margin we tested. The error bars represent the standard deviation of the maximum shear reading of a complete rotation. The shear interaction between the fluid and the bottom surface analysis is presented in the subsequent subsection.

### Fluid Interaction at Bottom Surface of the Flask

**Figure 10:**
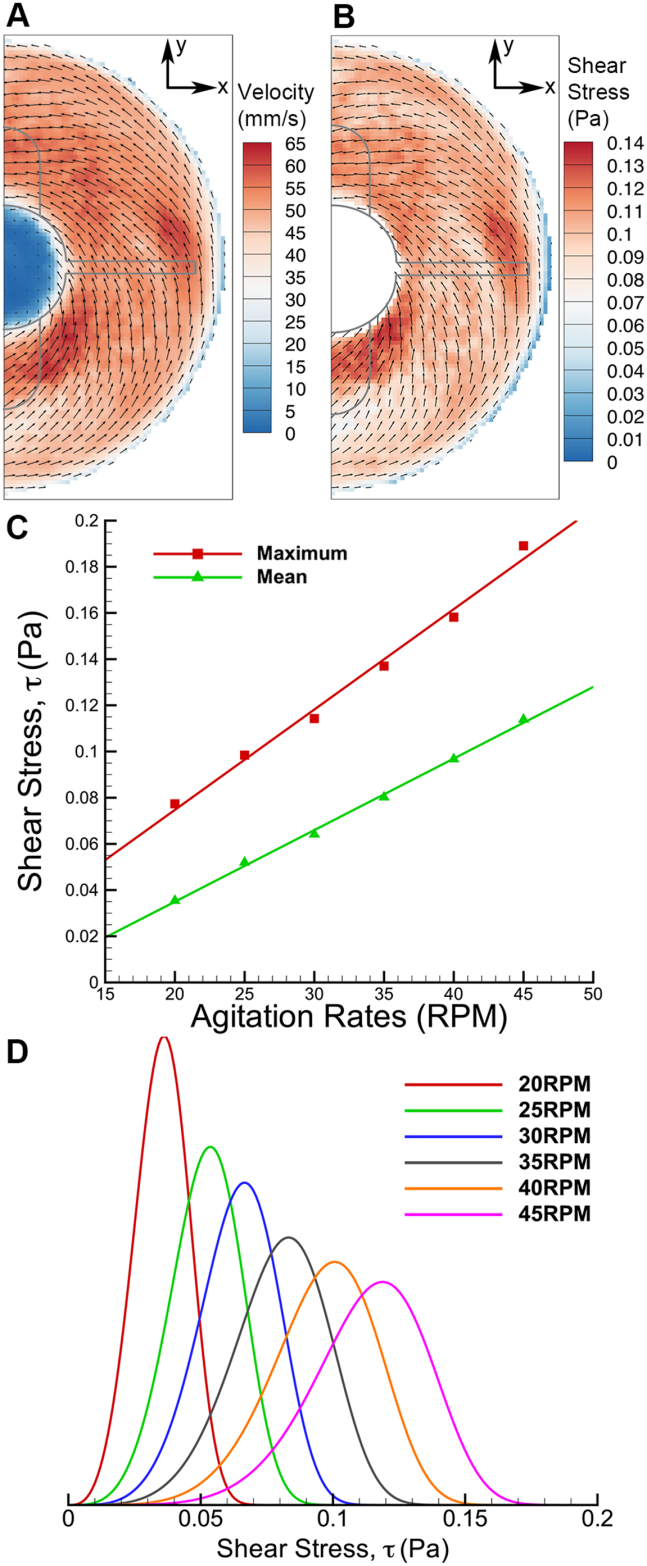
Results of fluid interaction at bottom surface of the flask. A) The velocity distribution at 40 RPM at z/H = 0.04. B) Shear stress near the bottom surface is higher than other azimuthal measurements. C) The mean and maximum shear stress increase linearly to the agitation speed. D) Shear stress distribution is skewed to higher shear magnitude at increasing speed.

In order to characterize the shear rate at the bottom surface, two measurements were conducted in azimuthal plane at height ratios of 0.04 and 0.06. Knowing zero velocity at z = 0, the gradient close to the bottom surface is calculated based on a parabolic fit between the velocity points at 3 different heights. Figure 10A shows the velocity vector at z/H = 0.04. Zero velocities at the center of the rotation are caused by the bump at the bottom of the flask. Furthermore, the vectors also show that the radial velocity component is more prominent than other vectors at higher planes, producing vortex behavior towards the center. The higher radially inward velocities are aligned with the results obtained in meridional imaging.

Although the velocity magnitude is lower than in other azimuthal plane measurements, the shear stress scale at the bottom surface is of significantly higher magnitude than in any previous measurements, as illustrated in Figure 10B and 10C. Unlike the shear stress in previous measurements that arises due to fluid-fluid interaction, the high magnitude of shear stress in Figure 10 is due to the fluid-wall interaction which produces higher shear gradient, resulting in higher shear magnitude. At higher spin rate, the shear distribution is also skewed to a higher value (Figure 10D).

### Mouse iPS Cells Proliferation

**Figure 11:**
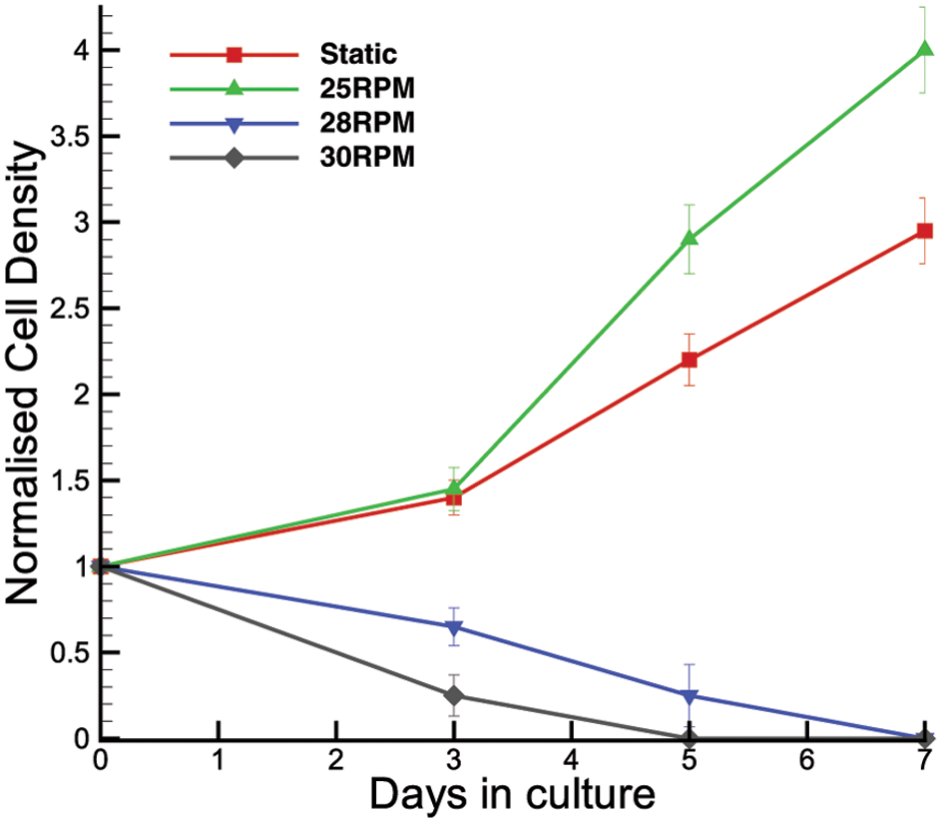
Normalized cell count over culture period for static and suspension culture method. A cell count achieved in the 20 RPM bioreactor was superior to the static culture over 7 days. The plot also shows that declining cell counts were obtained for 28 and 30 RPM suspension cell culture.

Figure 11 shows the normalized cell count density over the culture period grown in static culture, at 25 RPM, 28 RPM and 30 RPM agitation rates. It can be seen that the cells cultured at 28 RPM and above suffered an adverse effect on the cell count. Due to the fact that the density difference between Cytodex 3 microcarriers and water is minimal at 0.04 g/mL, it can be assumed that the microcarriers are neutrally buoyant and faithfully follow the fluid flow in the flask. Based on previous results, a spin rate of 28 RPM has its highest shear stress of 0.108 Pa with an average of 0.0592 Pa at the bottom surface of the flask. With declining cell count, it shows that mouse iPS cells are not able to maintain secure attachment to the microcarrriers at such a level of shear stress over the culture period. The plot shows that the highest cell count was obtained at 25 RPM, which translates to a maximum stress of 0.0984 Pa and mean of 0.0520 Pa. Such a flow condition was also able to maintain the cells’ pluripotency property [37]. This spin rate was demonstrated to produce a superior yield to the static culture. A low Reynolds number flow condition has a reduced mixing effect that could consequently produce counter-productive results to the cell count. The opposing effects of cell growth within a small shear range show that mouse iPS cells are very susceptible to shear condition. Details of cell morphology analysis can be found in Gupta et al. [33], [37]. Negligible suspension at speeds lower than 25 RPM was observed during the experiment. Hence, the culture condition at lower Reynolds number was comparable to static culture. Since the primary culture aim was long-term dynamic culture, 25 RPM was chosen as the minimum speed in the cell study. More experiments need to be conducted using different lines of cells to determine the optimal mechanical forces required to optimize the cell growth. For any rotational speed using a similar experimental configuration, this study provides a quantitative insight into the fluid dynamics in the spinner flask.

## Conclusions

In this study, we have shown extensive flow visualization in a spinner flask at various flow conditions. In the meridional plane point of view, the highest stress was recorded in the region downstream of the flat impeller. The velocity and shear varied linearly with Reynolds number. However, a significantly higher magnitude of shear was recorded in the azimuthal plane. Although higher velocities were obtained in a plane closer to the impeller, the highest velocity gradient occurs at the bottom surface of the flask. A notable value of shear was also present at the edge of the flat impeller. The distribution plots provide insights into how the margin of the mechanical force behaves at different Reynolds number.

It was found that the mouse iPS cells proliferated at their optimum condition at 25 RPM (Re = 1669) spinning rate. Flows at higher spin rates (and therefore Reynolds numbers) were not able to sustain positive growth of the cells. The higher shear stress in the flow had consequently detached the cells from the microcarriers, causing cell death and adverse cell count. On the other hand, at speeds below 25 RPM, the given setup showed that it was not able to maintain Cytodex 3 microcarrier beads in suspension. Hence, the minimum speed of 25 RPM was chosen since long-term dynamic suspension culture has been the primary aim in this study.

This study has shown that the use of spinner flask was able to increase cell yield compared to static culture. However, the homogeneous flow condition generated through the spinning impeller is at the expense of shear stress in the system. The cell experiment presented in this research served as an example in defining the shear stress condition specifically for mouse OG2 iPS cell culture. Additionally, the flow characterization defines the shear range within the spinner flask for future use.

Future work may include optimization studies for different cell lineages to determine their best culture condition. The results we have presented will enable one to acquire the shear stress distribution within the flask, thus creating a database of the required flow conditions for various cell types. The flow conditions for the cells can then be used as design requirements for the development of more efficient bioreactors. On the other hand, if the optimum biomechanical parameters for certain cell lineage are known, the required speed for the culture procedure can be determined based on the shear stress datasheet for the spinner flask. The flow mechanics knowledge would allow the required culture condition to be transferable to different types of bioreactor, thus reducing the experimental time spent in testing phase, should a different bioreactor needs to be utilized. However, flow characterization for various bioreactor types is required to define the shear margin in each design.

The small range of spin rates at which there was cell growth obtained in the cell culture experiment demonstrates that care should be taken in ensuring the impeller spins at an accurate speed in the cell culture. Furthermore, the use of a reliable and accurate system to drive the impeller will ensure consistency and repeatability, allowing exact replication to be performed by other research groups. This study provides insight into defining the optimum mechanical forces for cell culture procedures, thus providing quantitative parameters for optimizing bioreactor design.
